# Latent Class Cluster Analysis: Selecting the number of clusters

**DOI:** 10.1016/j.mex.2022.101747

**Published:** 2022-05-29

**Authors:** Olga Lezhnina, Gábor Kismihók

**Affiliations:** Leibniz Information Centre for Science and Technology University Library, Hannover, Germany

**Keywords:** LCCA, Model fit, Cluster separation, Stability of partitions

## Abstract

Latent Class Cluster Analysis (LCCA) is an advanced model-based clustering method, which is increasingly used in social, psychological, and educational research. Selecting the number of clusters in LCCA is a challenging task involving inevitable subjectivity of analytical choices. Researchers often rely excessively on fit indices, as model fit is the main selection criterion in model-based clustering; it was shown, however, that a wider spectrum of criteria needs to be taken into account. In this paper, we suggest an extended analytical strategy for selecting the number of clusters in LCCA based on model fit, cluster separation, and stability of partitions. The suggested procedure is illustrated on simulated data and a real world dataset from the International Computer and Information Literacy Study (ICILS) 2018. For the latter, we provide an example of end-to-end LCCA including data preprocessing. The researcher can use our R script to conduct LCCA in a few easily reproducible steps, or implement the strategy with any other software suitable for clustering. We show that the extended strategy, in comparison to fit indices-based strategy, facilitates the selection of more stable and well-separated clusters in the data.

• The suggested strategy aids researchers to select the number of clusters in LCCA

• It is based on model fit, cluster separation, and stability of partitions

• The strategy is useful for finding separable generalizable clusters in the data

## Graphical abstract

**Figure:**
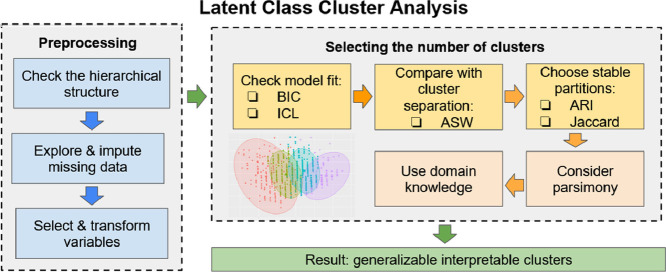

**Specifications table**

**Table:** 

Subject Area;	Psychology
More specific subject area;	Social Psychology
Method name;	Extended selecting strategy for LCCA
Name and reference of original method;	Fit indices-based selecting strategies for LCCA Marbac, M., & Sedki, M. (2019). VarSelLCM: An R/C++ package for variable selection in model-based clustering of mixed-data with missing values. *Bioinformatics, 35*(7), 1255–1257. https://doi.org/10.1093/bioinformatics/bty786 Petersen, K. J., Qualter, P., & Humphrey, N. (2019). The application of latent class analysis for investigating population child mental health: A systematic review. *Frontiers in Psychology, 10*, Article 1214. https://doi.org/10.3389/fpsyg.2019.01214
Resource availability;	The script in R (free downloadable software) is available on GitHub and in Supplementary Materials.

## Background and rationale

Latent Class Cluster Analysis (LCCA) is a clustering method for categorical variables with assumed multinomial distributions. LCCA belongs to model-based clustering methods, which fit probabilistic models to the data, in contrast to distance-based methods, which conduct partitions of observations based on a dissimilarity criterion [6]. In the frame of statistical analysis, LCCA (or Latent Class Analysis, LCA) is defined as an approach to modelling a discrete latent variable using multiple, discrete observed variables as indicators; in this paper, we consider LCCA exclusively from the clustering perspective, so that the discrete latent variable represents the cluster assignment. As a flexible tool, LCCA is the method of choice in many real-world circumstances, e.g., unequal covariance matrices, unequal numbers of observations in clusters, and poorly separated clusters [1] and therefore is increasingly used in psychological, social, and educational research [5].

Selecting the number of clusters in any clustering method, including LCCA, is a rather controversial topic [11]. Decisions on the number of clusters are inevitably subjective: clustering is “in the eye of the beholder” ([6], p. 65), as “true” clusters do not exist [14]. Therefore, the number of clusters in any clustering method is selected based on pre-specified criteria. For distance-based methods, these are dissimilarity criteria, such as the Average Silhouette Width (ASW). For model-based approaches, model fit indices are used, such as the Bayesian Information Criterion (BIC) or the Integrated Completed Likelihood (ICL) criterion. In LCCA, the BIC is frequently used as the single criterion, with the lowest value of the BIC indicating the best fitting model. Petersen et al. [21] reported that the BIC was the single criterion in majority of studies they analyzed, and Qiu & Malthouse [22] emphasized that the BIC was the only criterion implemented in commercial software, such as Latent Gold. The BIC, indeed, has a number of advantages over other information criteria [19], but it was shown that overreliance on the BIC as the single criterion could be detrimental for analysis [4], and an integrative approach to selection is required [[14], [20]]. Therefore, researchers include other criteria in their analysis. However, there is still no consensus on which criteria could be most useful for selecting the number of clusters in LCCA.

Most frequently, additional fit indices are applied, such as the ICL, which takes into account entropy and thus aims at finding well-separated clusters [2]. Marbac and Sedki [18] took an extension of the ICL called MICL (Maximum Integrated Complete-data Likelihood) for their implementation of LCCA in an R package. Flynt and Dean [8] supplemented their analysis with the elbow heuristic for the BIC plot. The elbow heuristic means finding the “elbow” of the plot, after which the change in successive values becomes less noticeable. This heuristic is effective and simple, and therefore typical for cluster analysis [3]. Meanwhile, other authors [1] applied the ASW, a criterion traditionally used for selecting the number of clusters in distance-based methods, to LCCA models, and showed that LCCA can perform at least as well in terms of the ASW as distance-based methods. It was shown that distance-based criteria employed in the frame of model-based clustering are useful for checking whether clusters have relatively small within-cluster dissimilarities [14].

A new strategy should go one step further than integration of model fit and cluster separation to include the bootstrap stability assessment [[7], [13]]. This procedure is typically conducted to check whether the chosen cluster solution depends on a specific dataset or can be generalized to new data [3]. For the final choice of the number of clusters, parsimony of a cluster solution, interpretability of clusters, and sizes of population shares should be taken into account [11,21].

In accordance with these considerations, an extended selecting strategy that we suggest involves assessing cluster solutions in terms of model fit and cluster separation and conducting the bootstrap assessment to select the most stable solution. The details of the strategy are outlined in the next section. Formulae of the BIC, the ICL, and the ASW, as well as a more detailed explanation of the stability assessment, can be found in the Additional Information section.

## Method details

### Preprocessing the data

Prior to LCCA, a few preprocessing procedures should be conducted that influence further analysis. Firstly, a hierarchical structure of the data needs to be explored to decide whether LCCA is sufficient, or multilevel LCA is needed. Then, missing data should be explored, and if necessary, the imputation procedures chosen. For imputation, we recommend the random forest algorithm, which was shown to be an effective and unbiased imputation method [10]; other analytical choices are also possible. Variable selection is an important step of the data preprocessing, but we do not dwell on different approaches here, as models in our illustration include all variables of interest. Normalization of variables is not required for LCCA, so this step, common for other techniques, can be omitted. Dichotomization of response options, although not infrequent in LCCA research [5], might be considered objectionable [16]. We recommend making a decision on dichotomization based on frequencies of response categories.

### Selecting the number of clusters

The extended selecting strategy for LCCA includes criteria based on model fit, cluster separation, and stability of partitions. Other considerations, such as parsimony, the size of population shares, and interpretability of clusters need to be taken into account for the final choice of the number of clusters.

In order to provide the researcher with detailed information on model fit and cluster separation, we wrote an easy-to-use custom function (LCCAselection) based on the VarSelClust function from the VarSelLCM package [18]. The function returns a data frame with information criteria and silhouette indices for one- to ten-cluster solutions. As visualization tools were shown to be important for deciding on the number of clusters [8,11], we included graphical output in the custom function to aid the cluster selection. The function produces a plot that integrates (i) the BIC plot for all cluster solutions to apply the elbow heuristic, (ii) the ASW plot for all cluster solutions, and (iii) vertical lines indicating the minimal BIC and the minimal ICL. Thus, the researcher can make informed decisions regarding model fit and cluster separation.

After two or three best solutions are chosen, their stability can be checked with another custom function (valfunc). The function accepts the data, the number of clusters, and the number of bootstrap samples as arguments to return the Jaccard coefficient and the adjusted Rand index (ARI) for bootstrap stability assessment of the cluster solution. The ARI and the Jaccard coefficient were chosen as they are two most widely used and easily interpretable metrics [12]; their formulae are given in the Additional Information section.

The most parsimonious cluster solution is preferable in case it satisfies other requirements, and clusters with excessively small population shares are considered inadequate regardless of the fit of the model [21]. Clusters should be interpretable from the perspective of domain knowledge of the researcher.

The selected clusters can be explored and visualised. In our R script, the researcher can find the item probability plot, the principal component analysis visualisation, the silhouette plot for clusters, and the barplot for the discriminative power of the variables. The discriminative power of the variables is defined as the logarithm of the ratio between the probability that the variable is relevant for the clustering and the probability that the variable is irrelevant for the clustering, given the best partition [18]. The greater value indicates that the variable is more important for the clustering.

## Method validation

To illustrate the strategy, we applied it (i) to simulated data with known cluster structure and (ii) to the data on teachers’ positive and negative views on information and communication technology (ICT) from the International Computer and Information Literacy Study (ICILS). With the simulated data example, we showed how model fit and cluster separation could be considered in terms of the trade-off between them. With the ICILS data, we provided end-to-end LCCA with the selection procedure. The data analysis was conducted with R, version 4.0.2 [23]. The R script (the *LCCA.R* and *LCCA_Simulated.R* files) is available at https://github.com/OlgaLezhnina/LCCA and in Supplementary Materials.

### Simulated data: model fit and cluster separation

**Fig. 1:**
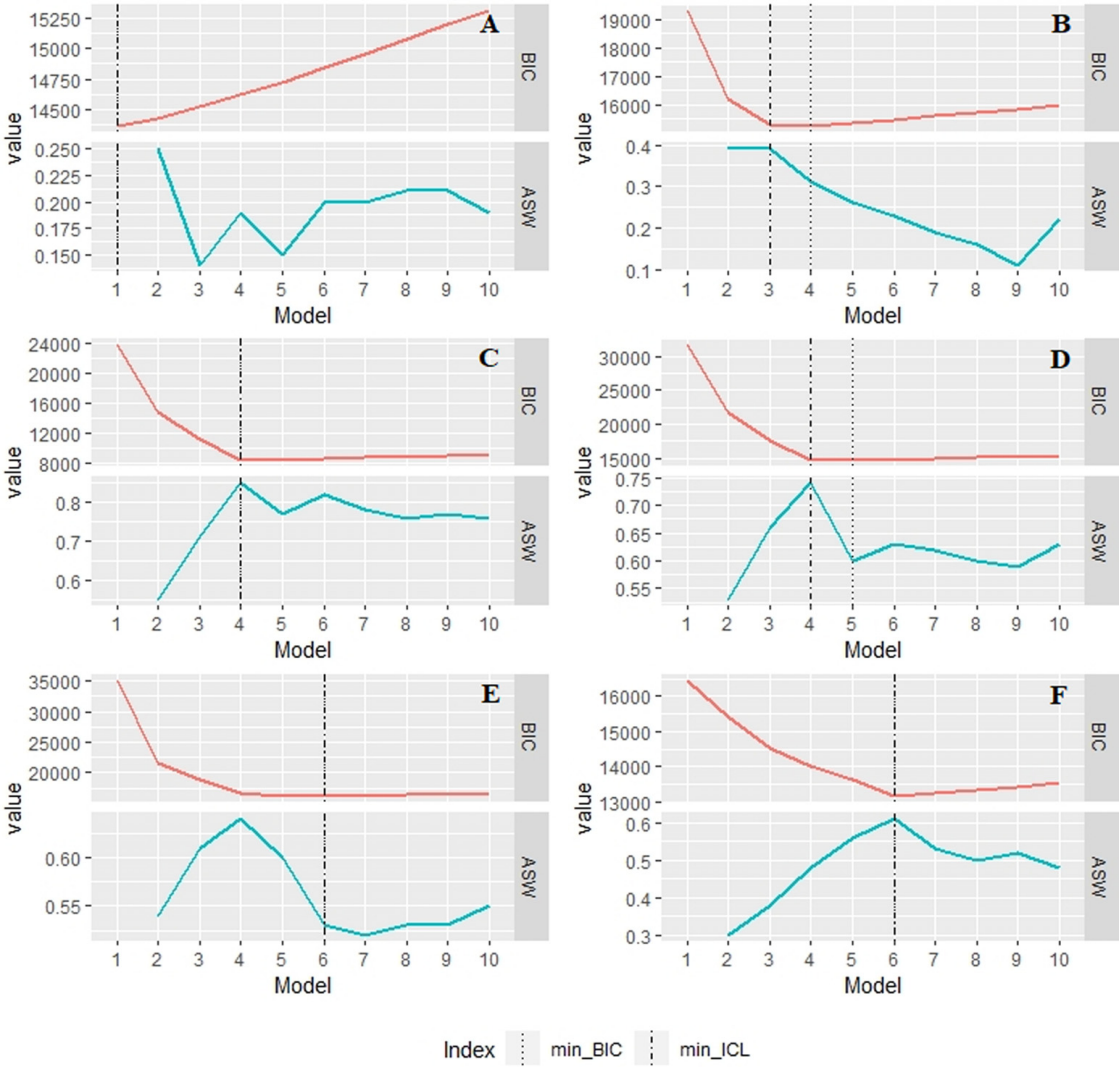
The graphic output of the LCCAselection function for six simulated datasets.

Firstly, we show the work of the strategy on simulated data. The ordinal clustered data was simulated with the clusterSim package [25]. The datasets contained the known structure of clusters. We generated three datasets with four clusters (N = 1550) and three datasets with six clusters (N = 2250), each with four response categories and six variables. The number of separable clusters in the datasets was varying (see Table 1). As the influence of the number of variables, the number of categories, sample size and unequal cluster sizes on LCCA performance was explored in large-scale simulation experiments [1], in our illustration we focused on cluster separation issues relevant to selecting the number of clusters. The clusters had unequal covariance matrices and unequal number of objects in them, which is typical for real-world data (for more information on the datasets, see the R script). We applied the LCCAselection function to the simulated datasets. The output of the function showing the fit indices and the ASW is presented in Fig. 1.

**Table 1:** Cluster selection results for the simulated datasets.

Dataset	Clusters	BIC	ICL	ASW	ARI	Jaccard
A	4/1	14622/-	−7351/-	.19/-	.05/-	.28/-
B	4/3	15252/15308	−7711/−7652	.31/.39	.80/.67	.76/.65
C	4/4	8316	−4109	.85	1	1
D	6/4	14752/14655	−7402/−7279	.63/.74	.61/.51	.52/.47
E	6/4	16141/16614	−8064/−8290	.53/.64	.91/.73	.86/.66
F	6/6	13148	−6521	.61	1	1

*Note*. BIC = Bayesian Information Criterion, ICL = Integrated Completed Likelihood criterion, ASW = Average Silhouette Width, ARI = Adjusted Rand Index. The number of clusters is given as total/separated, and the values of coefficients are given accordingly.

In Fig. 1 we see that the minimal BIC (vertical dotted lines in the plots) tended to indicate the “true” number of clusters in the data. The minimal ICL (dot-dashed lines) favoured well-separated clusters in datasets B and D, but not in dataset E. For well-separated clusters in datasets C and F, all criteria coincided in pointing at the correct cluster solution, and for the dataset A, LCCA was able to detect the problem of non-separated clusters in the data. The most interesting situations were presented by datasets B, D and E, in which the number of “true” clusters did not coincide with the number of separable clusters. The BIC elbow heuristic, together with the maximal ASW, indicated the number of separable clusters in all these datasets.

The values of the BIC, the ICL, the ASW, the ARI, and the Jaccard coefficient for each dataset (total/separated clusters) are given in Table 1. We can see how the trade-off between model fit and cluster separation works in LCCA: for dataset E, for instance, if we choose the minimal BIC and ICL solution, we will have the ARI = .91 and the ASW = .53, and if we prefer the BIC elbow solution, we will obtain better separated clusters with the ASW = .64 but the decrease in the ARI = .73. When the researcher aims for compact and well-separated clusters, the BIC elbow heuristics with the maximal ASW might be preferable to the minimal BIC value. Thus, the extended strategy is useful for finding well-separated clusters in the data.

### The ICILS data: End-to-end LCCA on teachers’ positive views dataset

The ICILS 2018 dataset (German sample) was retrieved from the International Association for the Evaluation of Educational Achievement (IEA) website [15] .^1^ The scores were on Likert scale from 1 (strongly agree) to 4 (strongly disagree). For our analysis, the positive views scores were recoded (reversed), so that higher scores represent more positive attitude to the ICT. Prior to the analysis, 57 rows with 100% missing variables were removed (.024 of the dataset). The resulting sample consisted of *N* = 2271 teachers from 182 German schools.

The hierarchical structure of the data was explored. Multilevel intraclass correlation coefficients for variables were from .002 to .039, and thus, non-multilevel methods could be used. Missing data (.01 of the dataset) was explored and visualized with the aggregation plot. Patterns of missingness that would imply that the data was missing not at random were not detected. Imputation was conducted with the random forest algorithm, and the resulting dataset was used for the further analysis. We did not select (or deselect) variables in the process of LCCA but included all variables of interest in the analysis and explored their relative importance. Frequencies of endorsements of different answer options for each item were explored. The extreme options (*strongly agree* and *strongly disagree*) were not underrepresented, and merging them with *agree* and *disagree* would lead to a substantial loss of information. Thus, it was preferable not to dichotomize the data.

**Fig. 2:**
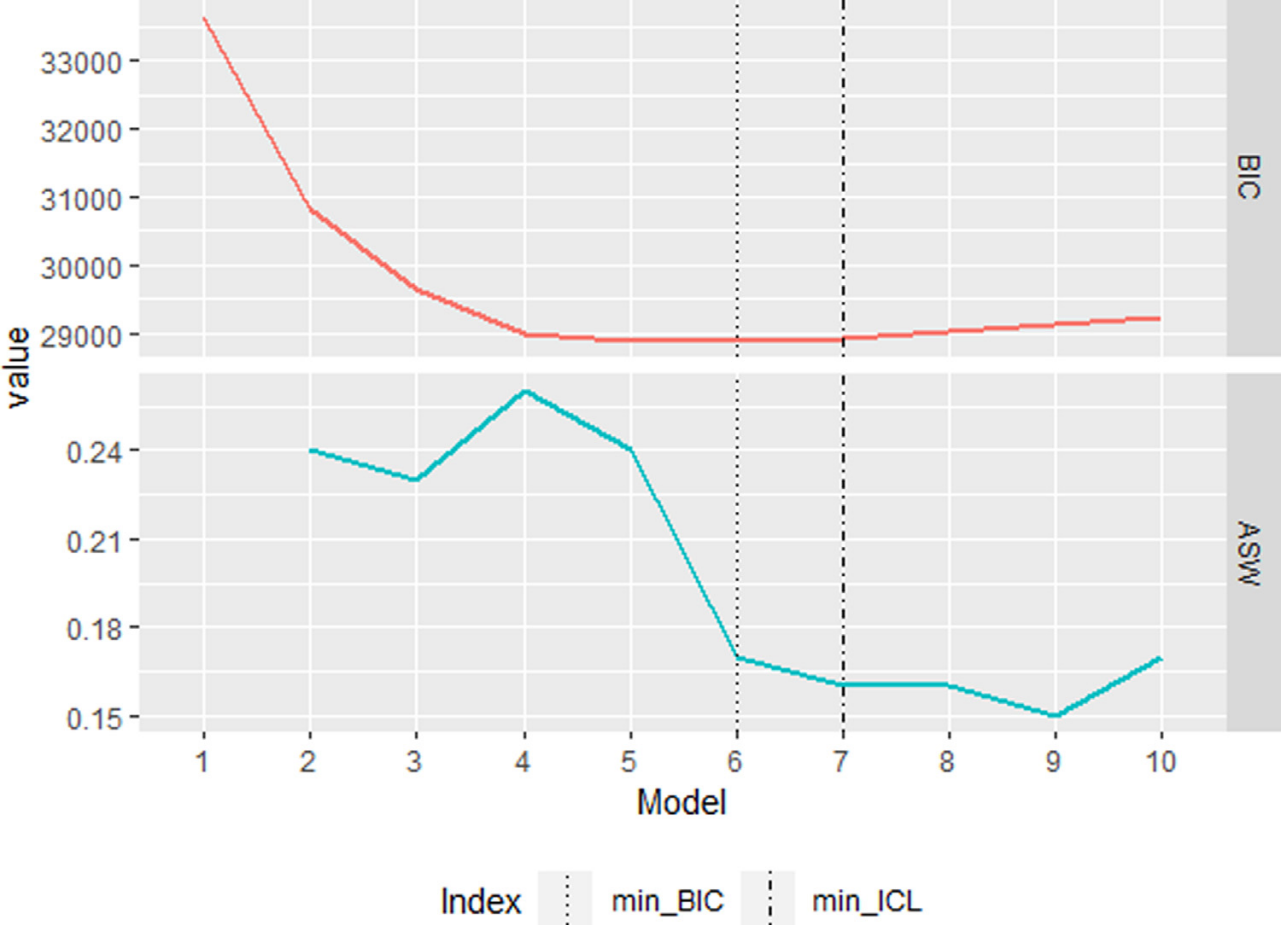
The graphic output of the LCCAselection function for the ICILS positive views dataset.

The custom function LCCAselection was applied to the positive views dataset and the negative views dataset to select the number of clusters. For the negative views dataset, all criteria indicated the four-cluster solution. Thus, we proceeded with the analysis of the positive views dataset and left further analysis of the negative views dataset for the interested reader. In Fig. 2, the graphical output of the LCCAselection function for the positive views dataset is presented. The BIC elbow heuristic and the maximal ASW pointed at the four-cluster solution, while the minimal BIC indicated the six-cluster solution. The minimal ICL, though, pointed at the seven-cluster solution.

The values of the criteria are reported in Table 2. It can be seen that for the seven-cluster solution indicated by the ICL, the ASW (.16) was lower than for other options we considered (.26 or .17). In addition to the parsimony considerations, it meant that we needed to reject the seven-cluster solution.

**Table 2:** Cluster selection results for the ICILS positive views dataset.

N clusters	BIC	ICL	ASW	ARI	Jaccard
1	33639.57	–16816.52	—		
2	30843.22	–15598.33	.24		
3	29648.88	–14998.36	.23		
4	28985.07	–14684.43	.26	.88	.85
5	28902.88	–14655.55	.24		
6	28880.90	–14693.07	.17	.76	.70
7	28925.91	–14651.72	.16		
8	29016.39	–14767.68	.16		
9	29115.62	–14736.12	.15		
10	29217.73	–14804.14	.17		

*Note.* BIC = Bayesian Information Criterion, ICL = Integrated Completed Likelihood criterion, ASW = Average Silhouette Width, ARI = Adjusted Rand Index.

The four- and the six-cluster solutions were compared in terms of their stability. The bootstrap stability assessment with 100 bootstrap samples was used. For the four-cluster solution, the ARI was .88 and the Jaccard coefficient .85, while for the six-cluster solution, the ARI was .76 and the Jaccard coefficient .70. Thus, the four-cluster solution was more stable. We calculated cluster population shares and discovered that the six-cluster solution had a very low population share in one of the clusters (.03 of the sample). Therefore, the more parsimonious four-cluster solution was selected as the final cluster model.

**Fig. 3:**
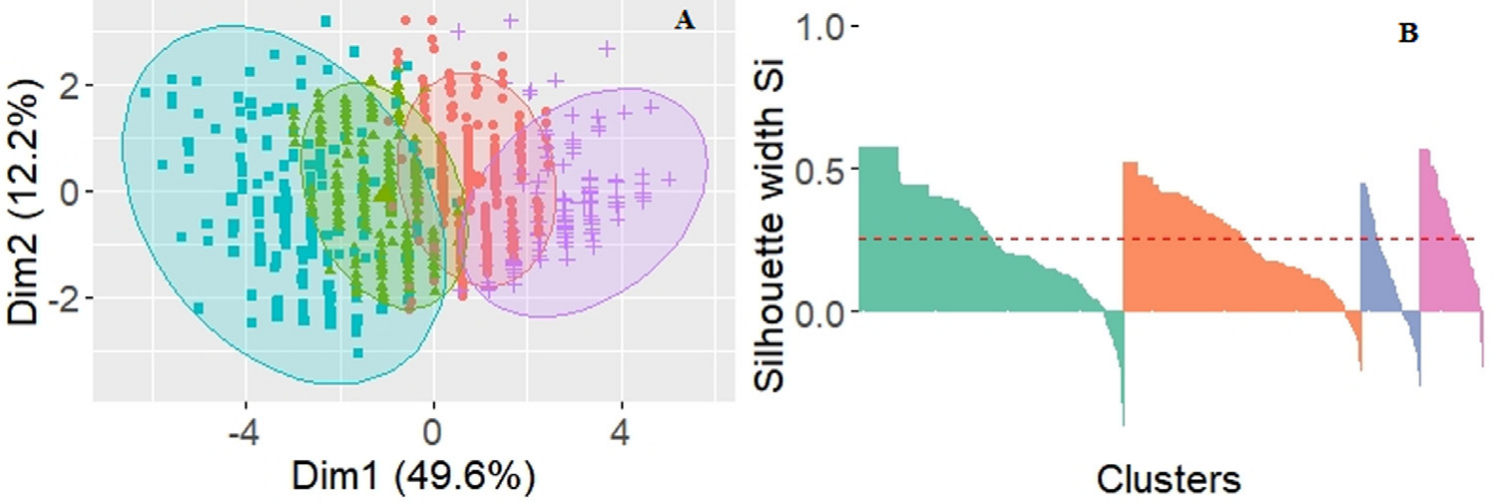
Cluster visualization and silhouette plot for the four-cluster solution.

In Fig. 3A, the results of principal component analysis for selected clusters are visualized. Such visualizations can be misleading, though, as more than two dimensions of the data are presented in the two-dimensional projection. The values of silhouette widths for all clusters, which are shown in Fig. 3B, are more reliable indicators of cluster separation (we can see that the clusters are still suboptimal in terms of separation).

**Fig. 4:**
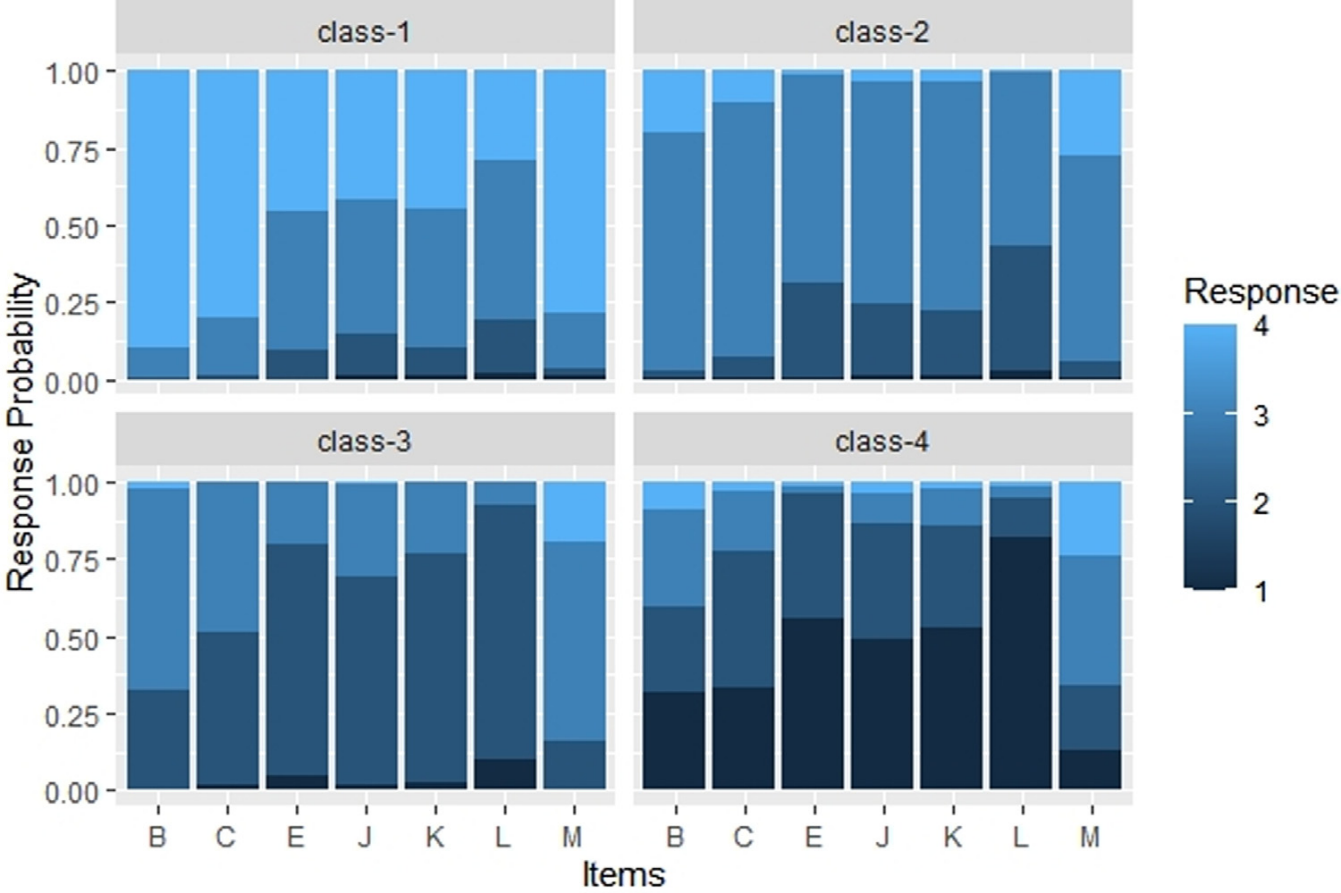
Item probability plot for the four-cluster solution.

The item probability plot for the selected solution is presented in Fig. 4. The order of the classes was changed to convey the ordinal information. The discriminative power of the variables was calculated (see the R script).

To summarise, we showed that the extended strategy is more comprehensive than strategies based on fit indices, such as the most commonly used BIC or the ICL. With the simulated data example, we showed that the combination of the fit indices and the ASW gives the clearest picture of the separable clusters. In case of the ICILS data, the strategy led to finding separable stable clusters, while overreliance on fit indices could have resulted in the choice of the six- or seven-cluster solution, which would be less beneficial in regard to cluster separation, and, more importantly, in regard to stability of partitions.

There are three levels of implementation of the strategy, so that researchers can either (i) rely on its conceptual background, or (ii) follow our recommendations on specific criteria and procedures, or (iii) use our R script in any way they find appropriate for their research goals. The limitation of the strategy is that both LCCA and the bootstrap stability assessment are computationally expensive, which might be inconvenient with very large datasets. In addition, we need to stress again that the quality of clustering is substantially influenced by variable selection, and this topic was not covered in the paper. We refer the interested reader to literature on variable selection.^2^

The extended strategy suggested in the paper widens the scope of tools for conducting LCCA. With a few easily reproducible steps, the researcher can select a cluster solution with optimal model fit, cluster separation, and stability of partitions. Thus, generalizable interpretable clusters can be more effectively found in the data.

## Declaration of Competing Interest

The authors declare that they have no known competing financial interests or personal relationships that could have appeared to influence the work reported in this paper.

## Appendix Supplementary materials

Image, application 1

Image, application 2

## Footnotes

1 To retrieve the data from the IEA website https://www.iea.nl/data-tools/repository/icils, it is necessary to agree to the terms and conditions associated with their use. For the German sample, see file *BTGDEUI2.sav*.

2 See Fop and Murphy [9], Maguire and Manuel [17], and Seo et al. [24]. In these papers, advantages and pitfalls of variable selection are outlined, well-performing algorithms (such as random forest and hidden Markov model on variables blocks) are discussed, and their implementations in R packages are suggested.
